# An update on hepatitis C virus genotype distribution in Jordan: a 12-year retrospective study from a tertiary care teaching hospital in Amman

**DOI:** 10.1186/s12879-019-4735-3

**Published:** 2019-12-31

**Authors:** Malik Sallam, Rawan Batarseh, Anas Natsheh, Jumana Abbadi, Esraa Al-Fraihat, Alaa’ Yaseen, Doaa Kaddomi, Nadia Khamees, Azmi Mahafzah, Gülşen Özkaya Şahin

**Affiliations:** 1 Department of Pathology, Microbiology and Forensic Medicine, School of Medicine, the University of Jordan, Queen Rania Al-Abdullah Street-Aljubeiha, /P.O. Box: 13046, Amman, 11942 Jordan; 2 Department of Clinical Laboratories and Forensic Medicine, Jordan University Hospital, Queen Rania Al-Abdullah Street-Aljubeiha, /P.O. Box: 13046, Amman, 11942 Jordan; 3 Department of Translational Medicine, Faculty of Medicine, Lund University, 22100 Malmö, Sweden; 4 Gastroenterology and Liver Division, Department of Internal Medicine, Jordan University Hospital, Amman, 11942 Jordan; 5 Department of Clinical Microbiology, Laboratory Medicine, Skåne University Hospital, 22100 Lund, Sweden

**Keywords:** Epidemiology, Genotype, Hepatitis C, HCV, Trend, Jordan

## Abstract

**Background:**

Nucleic acid hybridization (NAH) of hepatitis C virus (HCV) is a practical and reliable tool for virus genotyping. Genotype assignment is an important factor in the prediction of treatment success in chronic hepatitis C patients. The aim of this study was to determine the genotype distribution among HCV clinical isolates in Jordan between 2007 and 2018.

**Methods:**

Electronic and paper-based clinical data registry records from 2007 to 2018 at the Jordan University Hospital (JUH) were retrospectively examined for individuals with HCV genotype, HCV viral load, and alanine aminotransferase (ALT) testing results. Genotype determination was based on NAH technique using the HCV 5′ untranslated region (5′ *UTR*) with 386 requests available from 342 unique individuals.

**Results:**

A total of 263 out of 342 unique individuals (76.9%) had genotyping results available for final analysis with 259 individuals each having a single genotyping result. The most common HCV genotypes in the study were: genotype 4 (*n* = 142, 54.0%), genotype 1 (*n* = 87, 33.1%), genotype 3 (*n* = 16, 6.1%), genotype 2 (*n* = 9, 3.4%), other undetermined genotypes (*n* = 5, 1.9%) and mixed infections (*n* = 4, 1.5%). Sub-genotyping results were available for 46 individuals as follows: sub-genotype 4c/d (*n* = 13, 28.3%), sub-genotype 1a (*n* = 11, 23.9%), sub-genotype 1b (*n* = 10, 21.7%), sub-genotype 4a (*n* = 8, 17.4%), sub-genotype 3a (*n* = 2, 4.3%), sub-genotypes 2a/c and 4 h (n = 1, 2.2% for both). Individuals infected with genotype 1 showed higher viral load when compared to those infected with genotype 4 (*p* = 0.048, t-test). Younger HCV-infected individuals (< 52 years) had higher ALT levels compared to older individuals (*p* = 0.036, t-test). Self-reported risk factors for HCV acquisition included: history of previous surgery, invasive dental procedures, and blood transfusion, delivery at home, circumcision at home and wet cupping therapy (hijama).

**Conclusions:**

High genetic diversity of HCV was found in Jordan, with genotypes 4 and 1 as the most prevalent genotypes co-circulating in the country. Potential impact of virus genotype on disease markers (viral load, ALT) was detected and needs further assessment. The study can be helpful to plan for future prevention and management of HCV infection in Jordan.

## Background

Hepatitis C is considered a global health problem with about 71 million people living with chronic hepatitis C virus (HCV) infection and 399,000 mortalities each year (World Health Organization, 2018) [1]. The morbidity and mortality from HCV infection occur as a result of hepatic disease including fibrosis, cirrhosis, and hepatocellular carcinoma [2, 3].

HCV is an RNA virus that is characterized by high genetic variability [4]. It is classified into seven genotypes that are identified by 67 to 69% nucleotide sequence homology and are assigned with Arabic numerals [5, 6]. HCV genotypes are further split into sub-genotypes that differ by 20–25% in the nucleotide sequences and are assigned with small English letter suffixes after genotype (e.g. 1a, 2c, 4d, etc.) [5, 6].

The laboratory identification of HCV genotypes in clinical practice has a significant prognostic value for prediction of treatment success [7]. This is particularly evident for interferon-based treatment regimens with increasing evidence of its potential value in direct-acting antivirals (DAAs)-based therapy [8, 9]. In addition, sub-genotype determination appears to have an increasing clinical value in the era of DAAs [10]. However, treatment with new pan-genotypic regimens can be initiated without HCV genotyping results in certain situations (e.g. in areas where genotype determination is not available and/or not affordable [10]. Genotype determination through direct hybridization and probes directed against the 5′ untranslated region (5’*UTR*) of HCV, is considered a practical and reliable tool for HCV genotyping in clinical practice [11, 12].

The global distribution of HCV genotypes follows a characteristic geographic pattern [13]. Among all HCV genetic variants, genotype 1 represents the most prevalent genotype globally (46%), followed by genotype 3 (22%) and genotypes 2 & 4 (12% for each) [13, 14]. In the Middle East and North Africa (MENA) region, genotype 4 is the most prevalent owing to the large Egyptian HCV pool of infected individuals that is dominated by this genotype which is related to iatrogenic transmission roots dating back to 1950s [15–17]. However, genotype 1 appears to be ubiquitous in most countries of the region [13, 17].

Despite the lack of accurate data on HCV epidemiology in some countries of the MENA region, the few studies conducted in the region indicated that most MENA countries appeared to have low to moderate sero-prevalence of HCV [18].

In Jordan, the sero-prevalence of HCV appears to be relatively low (less than 0.5%) [19]. An updated report on the genotype distribution of HCV in Jordan is needed as scarce and outdated data are present from the country which focused on specific patient groups (i.e. hemodialysis patients) [20]. Solid and reliable epidemiologic data are needed to implement preventive strategies against HCV infection [21]. Thus, the aim of this study was to analyze genotype distribution of HCV among individuals with chronic HCV infection in Jordan in a large sample size and over a long study period.

## Methods

### Study population

HCV genotyping results at Jordan University Hospital (JUH) were available from routine clinical testing for genotyping of clinical isolates as a step in treatment consideration, with the majority of study subjects being treatment-naïve. The diagnosis of chronic hepatitis C at JUH relies on the presence of serologic evidence of infection with detection of the virus nucleic acid through nucleic acid amplification testing (NAT) for more than six months [22]. Hence, a fraction of the study population were negative for HCV NAT, likely representing past resolved infections. The study data were retrieved from electronic and paper-based clinical data registry of individuals attending JUH during the period 2007–2018. Patient data included age, gender, HCV genotyping request date, residence address (governorate), HCV viral load within one day of genotyping and alanine aminotransferase (ALT) level within one day of genotyping. In addition, limited data on self-reported possible risk factors for HCV acquisition were available from 18 individuals. In case of multiple intra-patient test requests, the data at the oldest date of collection was selected unless the later sample had sub-genotyping result. The concurrent or sequential detection of multiple HCV genotypes was defined as mixed infection.

### Ethical permission

The study was approved by JUH institutional review board (IRB/177/2019) in accordance with the Declaration of Helsinki. The IRB at JUH considered the use of data for the research in this study without the need for consent of the study subjects which would be impossible or impractical to obtain.

### HCV RNA extraction and reverse transcription

The extraction of HCV RNA was done using COBAS AMPLICOR Hepatitis C Virus Test, v2.0 during 2007–2015 and using QIAamp MinElute Virus Spin Kit (QIAGEN) during 2016–2018. The technical details of the procedures that were followed are described in (Additional file 1).

One-step HCV reverse transcription was done using COBAS AMPLICOR Hepatitis C Virus Test during 2007–2015 and HCV RNA Real Time Qualitative 2.0 (Nuclear Laser Medicine) during 2016–2018 following the manufacturer’s instructions in order to proceed to HCV genotyping.

### HCV genotyping

HCV genotyping was done using the Linear Array Hepatitis C Virus Genotyping Test v 2.0 (Roche Molecular Systems) based on reverse hybridization and targeting the 5’*UTR* solely during 2007–2015 and using GEN-C 2.0 Reverse Hybridization Strip Assay (Nuclear Laser Medicine) during 2016–2018, which discriminates between HCV genotypes on the basis of variations in the 5’*UTR* and *Core* genomic regions (2016–2018) following the manufacturer’s instructions.

### HCV RNA quantitation

The determination of HCV viral load was based on COBAS TaqMan HCV Test (Roche Molecular Systems) during 2007–2015 and using Xpert HCV Viral Load (Cepheid) during 2016–2018 (Additional file 1) [23, 24].

### Measurement of ALT

Testing of ALT levels in serum was done using Hitachi Modular platform (Roche) during 2007–2013 and Roche module Cobas 6000 (C-501) during 2014–2018 and the test kits were procured by Roche. Internal quality control checks were done on daily basis and external proficiency testing was started in 2018 and the unit of measurement of ALT was U/L.

### Statistical analysis

Statistical analysis was conducted through IBM SPSS Statistics 22.0 for Windows. Two-sided Fisher’s exact test (FET) and Mann-Whitney *U* test (M-W) were used when appropriate. To investigate association between the mean viral load and ALT with virus genotype, we used two-sided independent samples t-test. Temporal trends were analysed using two-sided linear-by-linear test for association (LBL). Statistical significance was considered for *p* < 0.050. The binomial distribution (Wilson score interval) 95% confidence interval (CI) of the prevalence was calculated using EpiTools epidemiological calculator available online (http://epitools.ausvet.com.au).

## Results

**Fig. 1:**
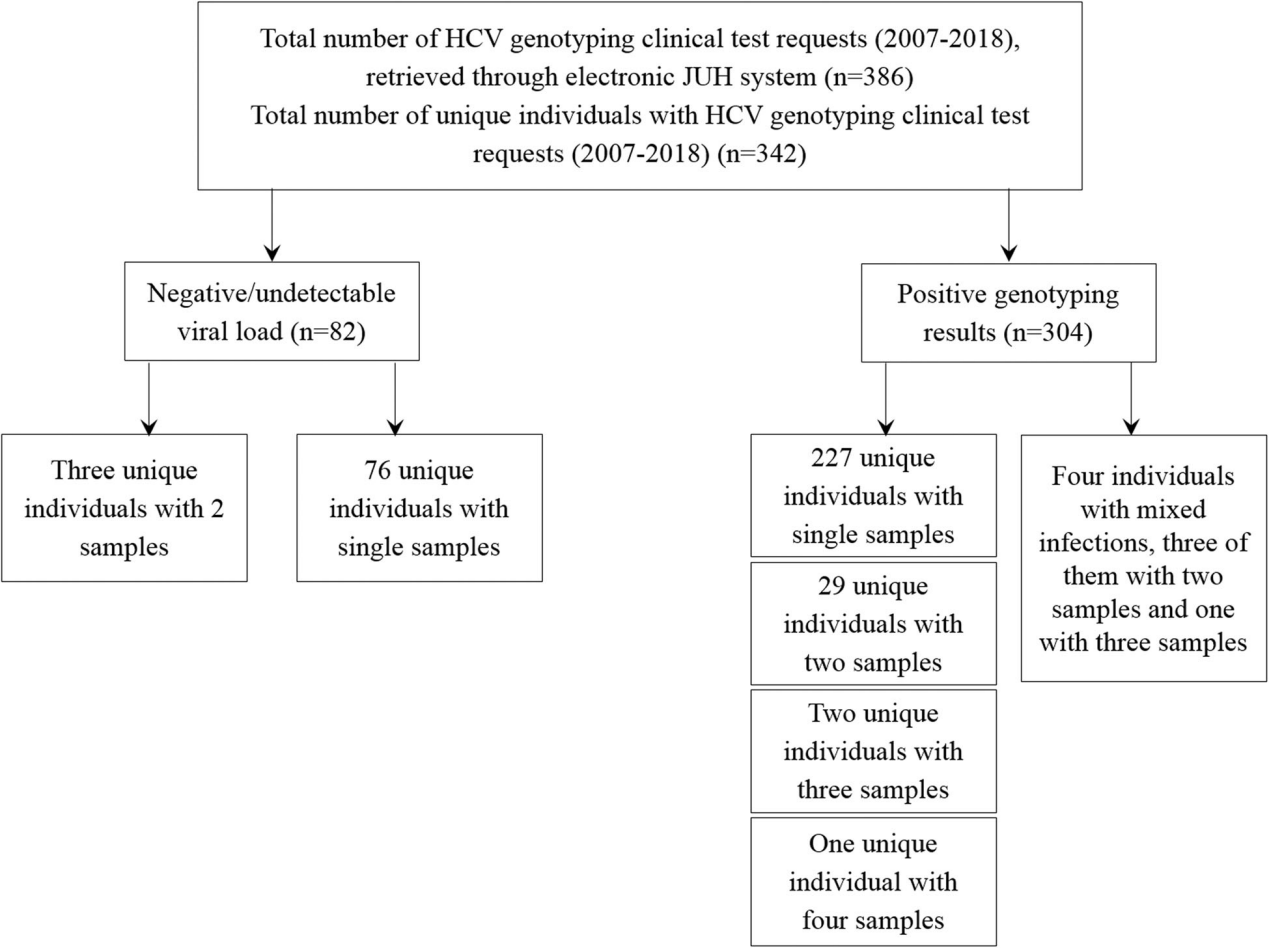
Flowchart indicating sample selection process for individuals and samples for subsequent HCV genotyping analysis. JUH: Jordan University Hospital, HCV: Hepatitis C virus. N: Number

The total number of HCV genotyping test requests during 2007–2018 was 386. Of those requests, 82 gave negative result (undetectable virus), of which six samples were taken from three unique individuals (two per patient). Of the positive samples, 227 were taken from unique individuals as single samples. Fifty-eight samples were taken from 29 unique individuals (two per patient), six samples from two individuals (three per patient) and one patient with four samples. Four unique individuals had the following special situations: one patient had dual infection with both genotype 1 and genotype 3 with the sample repeated twice (38-year-old male). For the other three specimens, sequential samples revealed different genotypes as follows: 49-year-old male patient with genotype 1 in 2007 and sub-genotype 2a/c in 2016, 48-year-old female with genotype 4 in 2011 and non-(1, 2, 3 or 4) in 2017 and 54-year-old with genotype 4 in 2015 and sub-genotype 1b in 2016 (Fig. 1). These four samples were neither included in the description of study subjects with genotyping results, nor in temporal trend analysis. This resulted in a total of 263 out of 342 unique individuals (76.9%), who had HCV genotyping results available for final analysis with 259 individuals each having a single genotyping result. The unique individuals with positive genotyping results were considered as the study population (*n* = 259).

The distribution of the study population was as follows: quarter (Q)1 (2007–2009): 88 individuals, Q2 (2010–2012): 67 individuals, Q3 (2013–2015): 50 individuals and Q4 (2016–2018): 54 individuals. About one-half of the study population were males (*n* = 130, 50.2%) and the median age of the individuals at time of genotyping was 52 years (mean: 49, interquartile range [IQR]: 38–60, Table 1). Gender-based comparison of age revealed that females were older than males (median age 54 vs. 49 years, IQR: [41–62] vs. [35–58] respectively, *p* = 0.045, M-W). The majority of individuals were living in Amman as an address (*n* = 142, 54.8%), followed by Balqa (*n* = 41, 15.8%) and Zarqa (*n* = 36, 13.9%). The vast majority of individuals were residents in the Central region of Jordan (*n* = 225, 86.9%), followed by individuals from the Southern region (*n* = 17, 6.6%) and five individuals were from the Northern region (1.9%) with data missing for 12 individuals. For the individuals with no HCV genotyping results, their median age at time of test request was 49 years (mean: 47, interquartile range [IQR]: 29–61), not statistically different from those with HCV positive genotyping results (*p* = 0.430, M-W). Gender distribution showed that females were more likely to have a negative result compared to males, but without statistical significance (*p* = 0.381, FET).

**Table 1:** Characteristics of the study population at Jordan University Hospital (2007–2018) stratified by HCV genotype

Characteristic	Total	1	2	3	4	Others^1^
	N^2^ (%)	N (%)	N (%)	N (%)	N (%)	N (%)
Total^3^	259 (100)	87 (33.6)	9 (3.5)	16 (6.2)	142 (54.8)	5 (1.9)
Age (median)	52	50	62	55	51	55
Gender						
*Male*	130 (50.2)	45 (51.7)	6 (66.7)	10 (62.5)	67 (47.2)	2 (40)
*Female*	129 (49.8)	42 (48.8)	3 (33.3)	6 (37.5)	75 (52.8)	3 (60)
Governorate						
*Irbid*	3 (1.2)	2 (2.3)	0	0	1 (0.7)	0
*Jerash*	2 (0.8)	1 (1.1)	0	0	1 (0.7)	0
*Balqa*	41 (15.8)	11 (12.6)	0	2 (12.5)	27 (19.0)	1 (20.0)
*Amman*	142 (54.8)	44 (50.6)	8 (88.9)	9 (56.3)	79 (55.6)	2 (40.0)
*Zarqa*	36 (13.9)	14 (16.1)	0	3 (18.8)	19 (13.4)	0
*Madaba*	6 (2.3)	3 (3.4)	0	0	3 (2.1)	0
*Karak*	7 (2.7)	3 (3.4)	0	0	3 (2.1)	1 (20.0)
*Tafilah*	5 (1.9)	1 (1.1)	0	0	4 (2.8)	0
*Ma’an*	2 (0.8)	1 (1.1)	0	0	1 (0.7)	0
*Aqaba*	3 (1.2)	2 (2.3)	0	0	1 (0.7)	0
*Unknown*	12 (4.6)	5 (5.7)	1 (11.1)	2 (12.5)	3 (2.1)	1 (20.0)
Quarter						
*Q1*^*4*^	88 (34.0)	27 (31.0)	6 (66.7)	8 (50.0)	44 (31.0)	3 (60.0)
*Q2*	67 (25.9)	21 (24.1)	2 (22.2)	3 (18.8)	41 (28.9)	0
*Q3*	50 (19.3)	16 (18.4)	0	3 (18.8)	31 (21.8)	0
*Q4*	54 (20.8)	23 (26.4)	1 (11.1)	2 (12.5)	26 (18.3)	2 (40.0)

^1^ Others: Other HCV genotypes include non-(1, 2, 3 or 4) genotypes; ^2^ N: number; ^3^ Total: The total number of unique individuals with positive HCV genotyping results. Four individuals with mixed infections were excluded from the description of the study subjects; ^4^ Q: Quarter

### Predominance of HCV genotypes 4 and 1 in Jordan

The distribution of HCV genotypes among unique individuals with positive genotyping results (*n* = 263), was as follows: genotype 4 (*n* = 142, 54.0%), genotype 1 (*n* = 87, 33.1%), genotype 3 (*n* = 16, 6.1%), genotype 2 (*n* = 9, 3.4%), other undetermined genotypes (*n* = 5, 1.9%) and mixed infections (*n* = 4, 1.5%). Characteristics of the study subjects stratified by HCV genotype are summarized in (Table 1). The study subjects infected with genotype 4 were marginally older than those infected with genotype 1 at the time of HCV genotyping request (median age: 51 vs. 50 years, *p* = 0.387, M-W). Additionally, the median age of the study subjects infected with genotype 1 was younger than individuals infected with all other sub-genotypes (50 vs. 53, *p* = 0.193, M-W).

Sub-genotyping results were available form 46 individuals with genotyping performed during the last quarter of the study. Sub-genotype distribution was as follows: 4c/d (*n* = 13, 28.3%), followed by 1a (*n* = 11, 23.9%), 1b (*n* = 10, 21.7%), 4a (*n* = 8, 17.4%), 3a (*n* = 2, 4.3%), 2a/c and 4 h (*n* = 1, 2.2% for both). The median age of the study subjects with sub-genotyping results was 55 years. Individuals infected with sub-genotype 1b were older than those infected with sub-genotype 1a (median age: 56 vs. 39 years, *p* = 0.072, M-W). In addition, the median age of the study subjects infected with sub-genotype 1a was younger than individuals infected with all other sub-genotypes (39 vs. 56, *p* = 0.047, M-W).

**Fig. 2:**
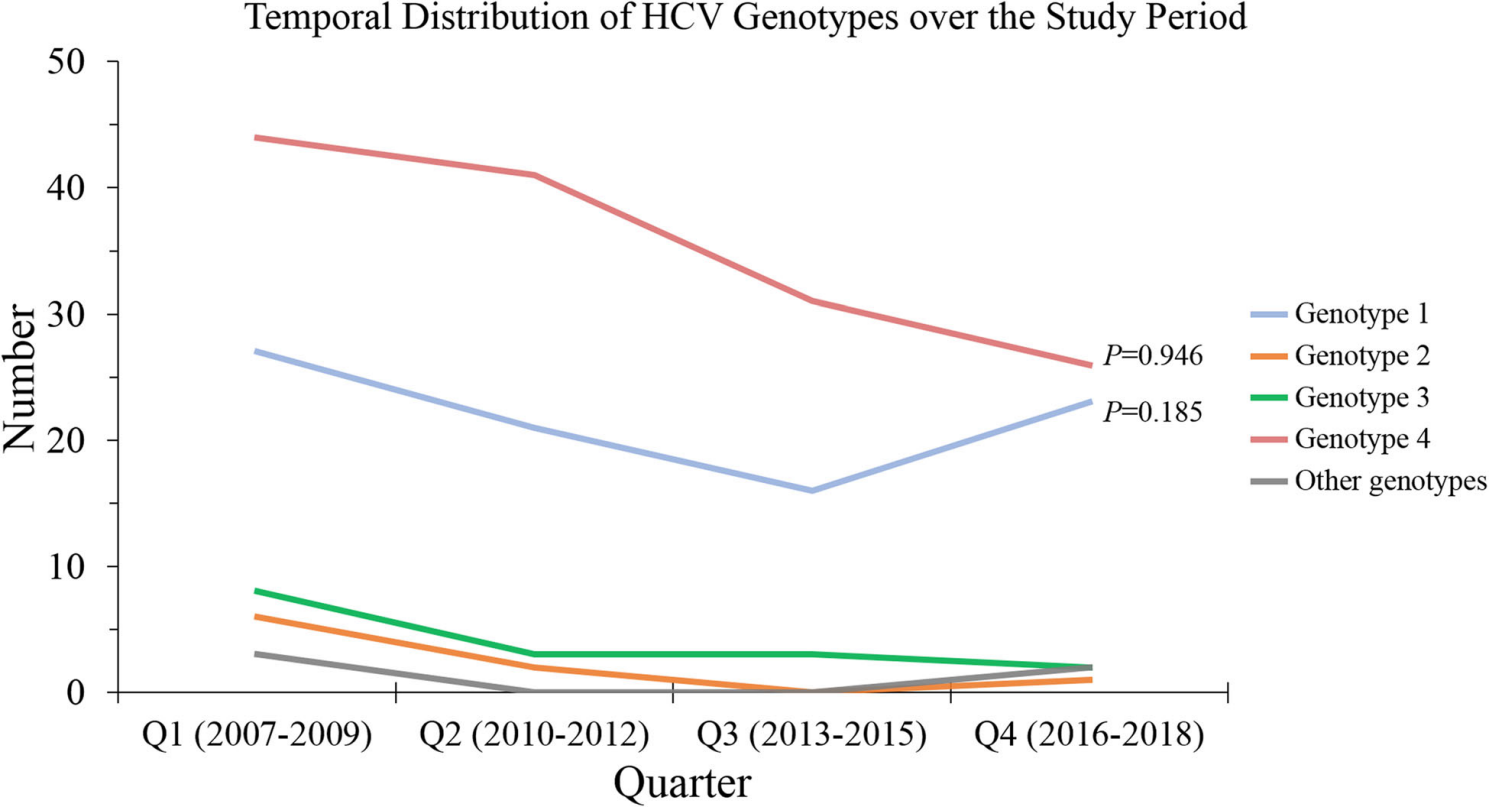
Temporal distribution of HCV genotypes over the quarters of the study period (2007–2018). Each quarter (Q) of the study period consisted of three years. *P* values were calculated using linear-by-linear test for association (LBL). Other HCV genotypes include non-(1, 2, 3 or 4) genotypes

**Fig. 3:**
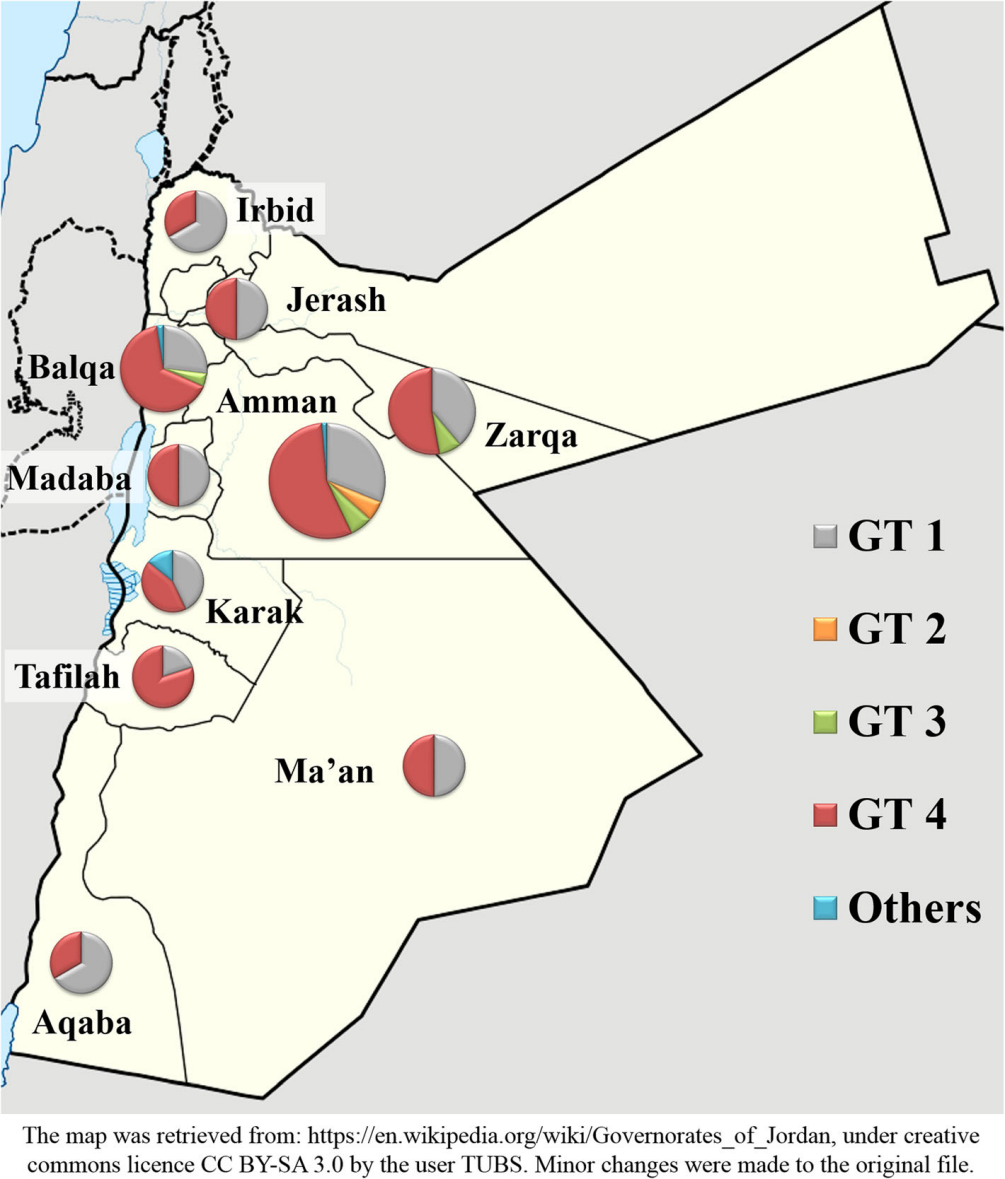
Distribution of HCV based on governorate of residence in Jordan (2007–2018). Other HCV genotypes include non-(1, 2, 3 or 4) genotypes. The map was retrieved from: https://en.wikipedia.org/wiki/Governorates_of_Jordan, under creative commons licence CC BY-SA 3.0 by the user TUBS. Minor changes were made to the original file

Upon tracking the temporal changes of the major genotypes over the study period (divided into quarters each of which consisted of three years), no statistically significant changes were found. Genotype 4 represented 50.0% of all HCV genotypes in Q1 and 48.1% in Q4 (*p* = 0.946, LBL, Fig. 2), whereas genotype 1 represented 30.7% of all HCV genotypes in Q1 and showed an increase to comprise 42.6% of all HCV genotypes in Q4 (*p* = 0.185, LBL, Fig. 2). In addition, genotype distribution across different governorates of the country did not show particular differences with genotype 4 as the most predominant genotype in the majority of regions (Fig. 3).

### HCV genotype association with viral load and ALT levels

Since HCV genotypes 4 and 1 were the most prevalent in this study, subsequent analysis was based on comparisons between these two genotypes. Viral load levels were available for 135 study subjects infected with HCV genotypes 4 and 1. The mean viral load level was significantly higher among individuals infected with genotype 1 compared to those infected with genotype 4 (2,005,177 vs. 902,308 IU/mL, *p* = 0.048, t-test). In addition, the mean viral load level was higher among females compared to males (1,384,386 vs. 1,177,421 IU/mL, *p* = 0.620, t-test), as well as among older individuals (equal to or more than 52 years) compared to younger individuals (less than 52 years) (1,313,276 vs. 1,260,437 IU/mL, *p* = 0.901, t-test).

For ALT levels, measurements were available from 139 individuals. The study subjects in the younger age group (less than 52 years) had a significantly higher mean ALT level of 71.9 U/L compared to older individuals (equal to or more than 52 years), who had a mean ALT level of 51.1 U/L (*p* = 0.036, t-test). Despite lacking statistical significance, the mean ALT level was higher among individuals infected with genotype 1 compared to those infected with genotype 4 (63.4 vs. 59.8 U/L, *p* = 0.736, t-test), and was also higher among males compared to females (65.3 vs. 56.5 U/L, *p* = 0.374, t-test).

### Possible risk factors for HCV acquisition in Jordan

Limited data were available regarding the possible routes for HCV acquisition among the study subjects. These data were available for 18 individuals who were inquired regarding the following possible risk factors: history of previous invasive surgery, history of invasive dental procedure, history of blood/blood product transfusion, use of contaminated needles, tattooing/piercing, birth at home for females, circumcision at home for males and hijama (wet cupping) therapy (Table 2). Seven individuals reported none of the aforementioned risk factors, while history of previous invasive surgery was reported by ten individuals. Three females reported history of previous invasive surgery, invasive dental procedure and birth at home altogether as possible risk factors for HCV acquisition. The median age for individuals who reported history of previous invasive surgery was older than the median age of the study population (62 vs. 52 years, *p* = 0.008, M-W). For genotype distribution, the study subjects with history of previous invasive surgery were infected with genotype 4 (60%, 4/10), genotypes 1 and 3 (2/10, 20% for both). For individuals with history of invasive dental procedure, the majority were infected with genotype 4 (6/7, 85.7%, Table 2).

**Table 2:** Possible self-reported risk factors for HCV acquisition in Jordan

ID^1^	Request Year^2^	Governorate	GT^3^	Risk Factor^4^					
				Surgery	Transfusion	Dental	Birth at home	Circumcision at home	Hijama^5^
JO_020	2007	Amman	1	Yes	No	No	No	No	No
JO_124	2010	Balqa	4	Yes	Yes	Yes	No	Yes	No
JO_131	2010	Amman	4	Yes	Yes	Yes	Yes	No	Yes
JO_138	2010	Amman	4	Yes	No	Yes	Yes	No	No
JO_153	2011	Amman	4	No	No	No	No	No	No
JO_154	2011	Amman	1	Yes	No	No	No	No	No
JO_155	2011	Irbid	1	No	No	No	No	No	No
JO_160	2011	Balqa	4	Yes	No	Yes	Yes	No	No
JO_161	2011	Amman	1	No	No	No	No	No	No
JO_172	2011	Zarqa	4	No	No	No	No	No	No
JO_189	2012	Amman	4	No	No	No	No	No	No
JO_197	2012	Zarqa	4	No	No	Yes	No	No	No
JO_198	2012	Amman	4	Yes	Yes	No	No	No	No
JO_201	2013	Madaba	4	No	No	No	No	No	No
JO_217	2013	Amman	3	Yes	Yes	No	No	No	No
JO_222	2013	Amman	3	Yes	Yes	Yes	No	No	Yes
JO_290	2016	Amman	4	Yes	Yes	Yes	Yes	No	No
JO_336	2017	Amman	Others	No	No	No	No	No	No

^1^ ID: Patient study number; ^2^ Request year: The year when HCV genotyping test was requested; ^3^ GT: Genotype, others: non-(1, 2, 3 or 4) genotype; ^4^ Risk factor: Self-reported risk factor for HCV acquisition. The study subjects were also asked about tattooing/piercing and history of contaminated needle exposure but none reported such risk factors; ^5^ Hijama: Wet cupping therapy

## Discussion

The current study was triggered by the lack of recent and accurate data assessing genotype distribution and risk factors for hepatitis C infection in Jordan. In the absence of an effective vaccine, prevention efforts and treatment strategies of hepatitis C rely largely on detailed depiction of epidemiologic aspects of the disease including better understanding of genotype and sub-genotype distribution, as well as identifying most-at-risk groups of individuals [21, 25–27]. Continuous evaluation of the epidemiology of hepatitis C is necessary as the global HCV epidemic is evolving rapidly [28]. This is related to improvements in treatment modalities and better health care conditions including safe blood transfusion and medical injection practices [27]. In addition, the political instability in some countries of the MENA region resulted in migration waves that might impact the epidemiology of various infectious diseases including hepatitis C in the region [29, 30].

Thus, the aim of this study was to assess the epidemiologic features of HCV infection in Jordan, using data collected over a 12-year period in a tertiary teaching hospital in Amman, with a bed-capacity of 550, which serves individuals from all regions of the country. The national coverage of the study was ensured by the presence of tested individuals residing in all regions of Jordan (Fig. 3). However, genotype distribution showed no particular differences across the major cities of the country.

The main finding of the study was the existence of two genotypes (4 and 1), which predominated HCV infections in Jordan over the last 12 years. Slightly more than 50% of chronic HCV infections in the study were attributed to genotype 4. The importance of this finding is related to the previous observation of less response to interferon therapy for both genotypes (4 and 1) [31]. The shift into use of DAAs to treat chronic HCV has become the standard-of-care and it should be considered for the majority of patients including treatment-experienced individuals and those with cirrhosis [32, 33]. Temporal trend analysis to track changes in genotype distribution did not reveal any changes over the last 12 years, since almost half of the cases were caused by genotype 4 in each quarter of the study period (Fig. 2). Nevertheless, a slight increase in the proportion of HCV infections caused by genotype 1 was found in the last quarter of the study. Furthermore, the younger age, higher viral load and higher ALT levels among individuals infected by genotype 1, particularly sub-genotype 1a, may hint to potential change in hepatitis C epidemiology in Jordan. This result necessitates continuous monitoring since genotype 1 has been linked with more aggressive disease [34, 35]. The older age of individuals infected with sub-genotype 1b might be related to older roots and links of this sub-genotype with health-care associated infections including previous history of invasive surgery or transfusion. This reasoning seems plausible considering the previous reports that have indicated similar links [34, 36, 37].

The observation of a higher mean viral load and ALT levels among the study subjects with genotype 1 infection compared to those with genotype 4 needs further elaboration. The common occurrence of two different HCV genotypes allows testing of possible association between virus genotype and some disease variables. Genotype 1 appeared to have a higher viral load compared to all other HCV genotypes in various studies from different global regions in agreement with our finding [38–40]. However, this result should be interpreted with caution considering the nature of the study population where genotyping was done without taking into consideration the natural history of HCV infection and variable time from infection till genotyping [41]. ALT is a serum marker used to assess liver injury leading to cirrhosis and higher level of this enzyme among individuals with genotype 1 infection could point to more severe liver damage compared to study subjects with genotype 4 infection [42]. A few studies investigated this issue with our results in line with findings of Riaz et al from a study conducted in Pakistan [43].

The co-circulation of multiple HCV genotypes with predominance of genotypes 4 and 1 has been reported from other Middle Eastern countries including Iraq, Kuwait, Palestine, Saudi Arabia, Syria and Yemen [17, 30, 44, 45]. Possible explanation of this observation can be related to the presence of a large number of Egyptian residents in Jordan comprising about 6.7% of the total residents in the country as of 2015 [46]. This pattern has also been reported in the Gulf Cooperation Council countries of the MENA with possible association of this observation to expatriate populations reflecting the HCV epidemics in their countries of origin [47]. For North African countries, the HCV genotype distribution is different with the predominance of genotype 1 among most countries, except in Egypt and Sudan where genotype 4 predominates almost solely [15, 48].

Sub-genotype analysis revealed a large genetic diversity of HCV in Jordan with sub-genotypes 4c/d, 1a and 1b being the most common. This diversity is likely pointing to multiple introductions of HCV into the country over a long period. However, this hypothesis needs further evaluation using phylogenetic analysis of molecular HCV sequences to achieve more robust conclusions about the evolutionary history of the virus in Jordan.

Despite the limited data on possible risk factors for HCV acquisition, a few observations have been made. Previous history of invasive surgery and invasive dental procedures were the most common risk factors associated with HCV acquisition in the study subjects. The older age of individuals with previous history of surgery might point to better compliance to universal precautions over time in Jordan resulting in lower transmission among the younger individuals undergoing invasive surgical procedures. In addition, invasive dental procedures are known as potential risk factors for blood-borne virus acquisition including HCV and was previously reported as a risk factor for hepatitis B infection in Northern Jordan [49, 50]. This should be considered in the implementation of robust infection control guidelines and increasing awareness among health-care workers involved in dental care. Moreover, wet cupping (hijama) therapy and delivery at home were also among the risk factors reported by a few study subjects. Wet cupping therapy is a form of ancient alternative medicine that is still practiced in the MENA region [51]. It involves drawing blood by vacuum from skin incisions. Since these practices are often done by unprofessional individuals with lack of safety precautions and disposal or re-use of sharps, it may have been a contributing factor to a fraction of HCV infections reported in the study [52].

Preventive strategies as a step for hepatitis C eradication have been reviewed by Hesamizadeh et al and included targeting most-at-risk groups with focused preventive measures, and the promotion of awareness about HCV infection among the public [25]. All these factors should be considered by the local health authorities in Jordan focusing on the aforementioned possible routes of HCV transmission for successful prevention efforts.

The concurrent detection of multiple HCV genotypes (or distinct genetic strains) was previously reported with variable prevalence estimates depending on the studied populations (e.g. injection drug users, hemodialysis patients, etc.) [53–55]. These mixed infections might increase the risk of treatment failure [56]. A single individual in our study was found to harbour a mixed infection with genotypes 1 and 3 on three different occasions, which makes this result credible yielding a prevalence of mixed HCV infection in the current study of 0.38% (95% CI: 0.07–2.12%). This result might reflect an underestimation of the true prevalence of mixed HCV infections in Jordan, considering the method used for genotyping. Re-infection or the appearance of previously undetected mixed infection was found in three individuals (1.14, 95% CI: 0.39–3.30%) which might indicate the presence of a minority population with high risk behaviour or point to minority HCV populations that emerged following the treatment of a previously dominating strain [57].

Several limitations in this study were inevitable and included sampling bias. Sampling was based on the individuals who were considered to be treated, therefore, the true distribution of HCV genotypes in the country might be slightly different. In addition, two different methods for determining HCV genotypes were used and thus, the availability of sub-genotyping results were present only in fraction of individuals during the latest quarter of the study period. Moreover, lack of detailed individual data on risk factors, nationality and data on treatment history precluded further assessment of the HCV epidemic in this study. Finally, the vast majority of the study subjects were residing in Central Jordan. However, this issue might have little effect on the study results considering that about two-thirds of Jordan population reside in this region. All these limitations should be considered together with utilization of molecular methods to prospectively assess the epidemiology of HCV in Jordan in future projects.

## Conclusions

The current report represents an update on genotype distribution of HCV in Jordan among individuals with chronic infection who were considered for treatment. The inclusion of individuals residing in different regions of the country can give clue to the national status of genotype distribution. Genotype 4 predominated the cases followed by genotype 1, which highlights the importance of implementing newer treatment and preventive strategies considering the poor response of both genotypes to traditional therapies. Large genetic diversity of the virus was observed which indicates the possibility of multiple introductions of HCV over a long period of time. However, this should be further investigated using molecular epidemiology tools to assess the dynamics of transmission and sub-genotype contribution to the local and regional HCV epidemics. Most-at-risk groups and risk factors for HCV acquisition should be considered by the local health authorities in Jordan with focused intervention measures to limit the spread of infection in the country.

## Supplementary information

**Additional file 1:** Detailed methods.

## Data Availability

Not applicable.

## Abbreviations

ALT: Alanine aminotransferase
CI: Confidence interval
FET: Two-sided Fisher’s exact test
HCV: Hepatitis C virus
IQR: Interquartile range
JUH: Jordan University Hospital
MENA: Middle East and North Africa
M-W: Mann-Whitney *U* test
