# Supplementary material for: An update on hepatitis C virus genotype distribution in Jordan: a 12-year retrospective study from a tertiary care teaching hospital in Amman
Source: BMC Infect Dis. 2019 Dec 31;20:3. doi: 10.1186/s12879-019-4735-3 (PMC6938611; doi:10.1186/s12879-019-4735-3)
Supplement: Supplementary file 1 — Additional file 1: Detailed methods. [file 12879_2019_4735_MOESM1_ESM.pdf]

An update on hepatitis C virus genotype distribution in Jordan: a 12-year retrospective study from a tertiary care teaching hospital in Amman. Sallam M. *et al*.

Additional File 1

Detailed Methods

HCV RNA extraction and reverse transcription

The extraction of HCV RNA was done using COBAS AMPLICOR Hepatitis C Virus Test, v2.0 during 2007-2015 and using QIAamp MinElute Virus Spin Kit (QIAGEN) during 2016-2018. For COBAS AMPLICOR Hepatitis C Virus Test, the procedure was as follows: 200 µL of serum was added to 400 µL lysis solution (containing 6 µL of internal control) and the mixture was incubated at 60 °C for 10 minutes. This was followed by adding 600 µL of 99.9% isopropyl alcohol. The incubation step was done at 25°C for 5 minutes followed by centrifugation at 12,000 *g* for 15 minutes. A total of 1000 µL of freshly prepared 70% ethanol was added to the pellet followed by centrifugation at 12,000 *g* for 5 minutes. Finally, a total of 200 µL specimen diluent was added to 50 µL pelleted sample. For the QIAamp MinElute Virus Spin Kit the procedure was as follows: For manual procedure, 200 µL of serum was added to 25 µL QIAGEN Protease and 200 µL Buffer AL (containing 28 µg/mL of carrier RNA) and the mixture was incubated at 56 °C for 15 minutes. This was followed by adding 250 µL 99.9% ethanol and incubating for 5 minutes at 25°C. This mixture was then applied to QIAamp MinElute column and centrifuged at 6000 *g* for 1 minute. Washing step was done by adding 500 µL of Buffer AW1 followed by centrifugation of the column at 6000 *g* for 1 minute. This was followed by adding 500 µL of buffer AW2 and centrifugation of the column at 6000 *g* for 1 minute, followed by adding 500 µL 99.9% ethanol and centrifugation of the column at 6000 *g* for 1 minute. After that, columns were centrifuged at 20,000 *g* for 3 minutes. Drying of filter membrane was done by opening spin column lid at 56 °C for 3 minutes. Finally, elution of

RNA was done by adding 60 µL buffer AVE into each column with incubation for 1 minute at 25 °C followed by centrifugation at 20,000 *g* for 1 minute. For automated procedure, 200 µL of serum was loaded in fully automated QIAcube (QIAGEN) using the standard protocol for purification of viral nucleic acids from plasma or serum. The final elution volume was 60 µL.

One-step HCV reverse transcription was done using COBAS AMPLICOR Hepatitis C Virus Test during 2007-2015 and HCV RNA Real Time Qualitative 2.0 (Nuclear Laser Medicine) during 2016-2018 following the manufacturer's instructions in order to proceed to HCV genotyping.

HCV RNA Quantitation

The determination of HCV viral load was based on COBAS TaqMan HCV Test (Roche Molecular Systems) during 2007-2015 and using Xpert HCV Viral Load (Cepheid) during 2016-2018. The starting volume of patient serum was 500 µL. Following reverse transcription of the HCV target RNA, COBAS TaqMan 48 Analyzer was used for performing real-time PCR according to the manufacturer's instructions with linear range of the assay between 25 IU/mL and 3.91×10^8 IU/mL. For the Xpert HCV Viral Load assay, 1.2 mL of serum was added to a test cartridge and the cartridge was loaded into GeneXpert Instrument for direct nucleic acid purification and real-time reverse transcriptase PCR with linear range of assay between 10 IU/mL to 1.00×10^8 IU/mL.
